# A metal-electrode-free, fully integrated, soft triboelectric sensor array for self-powered tactile sensing

**DOI:** 10.1038/s41378-020-0154-2

**Published:** 2020-08-10

**Authors:** Lingyun Wang, Yiming Liu, Qing Liu, Yuyan Zhu, Haoyu Wang, Zhaoqian Xie, Xinge Yu, Yunlong Zi

**Affiliations:** 1 Department of Mechanical and Automation Engineering, The Chinese University of Hong Kong, Shatin, N.T., Hong Kong SAR, China; 2 Department of Biomedical Engineering, City University of Hong Kong, Kowloon, Hong Kong SAR, China; 3 Department of Applied Biology and Chemical Technology, The Hong Kong Polytechnic University, Hung Hom, Hong Kong SAR, China; 4 State Key Laboratory of Structural Analysis for Industrial Equipment, International Research Center for Computational Mechanics, Department of Engineering Mechanics, Dalian University of Technology, Dalian, 116024 China

**Keywords:** Engineering, Sensors

## Abstract

The dramatic advances in flexible/wearable electronics have garnered great attention for touch sensors for practical applications in human health monitoring and human–machine interfaces. Self-powered triboelectric tactile sensors with high sensitivity, reduced crosstalk, and simple processing routes are highly desirable. Herein, we introduce a facile and low-cost fabrication approach for a metal-electrode free, fully integrated, flexible, and self-powered triboelectric tactile sensor array with 8-by-8 sensor units. Through the height difference between the sensor units and interconnect electrodes, the crosstalk derived from the electrodes has been successfully suppressed with no additional shielding layers. The tactile sensor array shows a remarkable sensitivity of 0.063 V kPa^–1^ with a linear range from 5 to 50 kPa, which covers a broad range of testing objects. Furthermore, due to the advanced mechanical design, the flexible sensor array exhibits great capability of pressure sensing even under a curved state. The voltage responses from the pattern mapping by finger touching demonstrate the uniformity of the sensor array. Finally, real-time tactile sensing associated with light-emitting diode (LED) array lighting demonstrates the potential application of the sensor array in position tracking, self-powered touch screens, human–machine interfaces and many others.

## Sensors: Fully integrated, soft sensor array for self-powered tactile sensing

A fully integrated soft tactile sensor array (ISTSA) that is self-powered and without metal electrodes has been developed using a low-cost, easily performed fabrication process. A sensor array is a group of sensors used for collecting and processing electromagnetic signals, and arrays of tactile sensors have recently attracted substantial attention with the growth of wearable electronics and application in health monitoring and human-machine interfaces. A team headed by Yunlong Zi at The Chinese University of Hong Kong succeeded in developing an ISTSA with remarkable sensitivity. The ISTSA is so flexible that it exhibits excellent pressure sensing even in a curved state. The authors conducted real-time tactile sensing with LED lighting and finger touching, and they believe their ISTSA has considerable potential for application in such areas as position tracking, self-powered touch screens, and wearable electronics.

## Introduction

Recently, the rapid development of the advanced technology of flexible/wearable electronics has enabled a variety of applications in electronic skins and human–machine interfaces^1–8^. In particular, tactile sensors capable of transducing physical touch to electrical signals have demonstrated their practical application in human health monitoring, security monitoring, and artificial intelligence^9–15^ based on different transduction mechanisms, including capacitance^16–18^, piezoresistivity^19–21^, and piezoelectricity^22,23^. Owing to the advantages of high sensitivity, low cost, diverse material selection, and zero power consumption, another type of sensor based on a triboelectric nanogenerator (TENG), with the sensing mechanism of coupling triboelectrification^24–27^ and electrostatic induction^28^, has aroused great interest among researchers^29–32^. For example, Wang et al. ^31^ reported a self-powered triboelectric sensor matrix composed of polydimethylsiloxane (PDMS) as an electrification layer and patterned Ag electrodes serving as charge-sensing components and circuit connections. This sensor matrix has a resolution of 5 dpi and a pressure sensitivity of 0.06 kPa^–1^ and is capable of real-time tactile mapping. Further, the same group developed a self-powered tactile sensor with high stretchability and transparent patterned Ag nanofiber electrodes^32^. Additionally, a graphene-based self-powered touch sensor with atomically thin graphene (<1 nm) as the electrode and PDMS as the electrification layer was reported by Lee et al.^30^. The auxetic mesh design endows the touch sensor with the capability to maintain a stable electrical output while being stretched. These emerging triboelectric tactile sensors exhibit attractive features; however, the fabrication processes involve either specialized equipment or high-cost metal electrodes, which complicates the fabrication process and increases the cost for large-scale production. Thus, simple fabrication and economic processes are highly desirable. Meanwhile, hydrogel/ionogel, another promising type of conductor owing to its merits of high conductivity, transparency, and stretchability, has been widely explored in applications for touch panels^33^, sensors^34–36^, TENGs^37–39^, and soft robotics^40^.

In addition, another issue generally associated with triboelectric-type sensor arrays is the crosstalk between sensor units as well as the electrodes^41,42^, arising from electrostatic induction, which significantly limits their practical application for precise position identification in response to contacting objects. Thus, great efforts have been devoted to reducing this useless crosstalk, such as introducing a dielectric shielding layer^32,43,44^ or metal screening layer^45^. Nevertheless, more involved layers not only complicate the fabrication process but also may compromise the stretchability/flexibility of the device due to mismatch of the elastic modulus of different layers.

Herein, to address the abovementioned issues, we present a facile, low-cost process to fabricate a metal-electrode-free, fully integrated, soft triboelectric sensor array (ISTSA), which is composed of an elastomer (Ecoflex) as the electrification layer and a gel state of polyvinyl alcohol/polyethyleneimine (PVA/PEI) sealed as the sensor units and electrodes. In particular, without the shielding layer, this ISTSA suppresses the crosstalk arising from the electrodes by introducing a height difference between the sensor units and serpentine electrodes. Based on a single-electrode TENG mode, the output sensing signals of an individual sensor unit regarding various contact objects, pressure sensitivity, different mechanical stimuli, and long-term stability were investigated. Further, the crosstalk derived from the electrodes to the adjacent sensor units was illustrated using real-time sensing, with the signal-to-noise ratio larger than eight-fold. The uniformity of the ISTSA was demonstrated by pattern mapping through finger touching. Finally, light-emitting diode (LED) array lighting by touching the corresponding sensor unit demonstrates the potential application of the ISTSA for position tracking and human–machine interfaces.

## Results and discussion

**Fig. 1:**
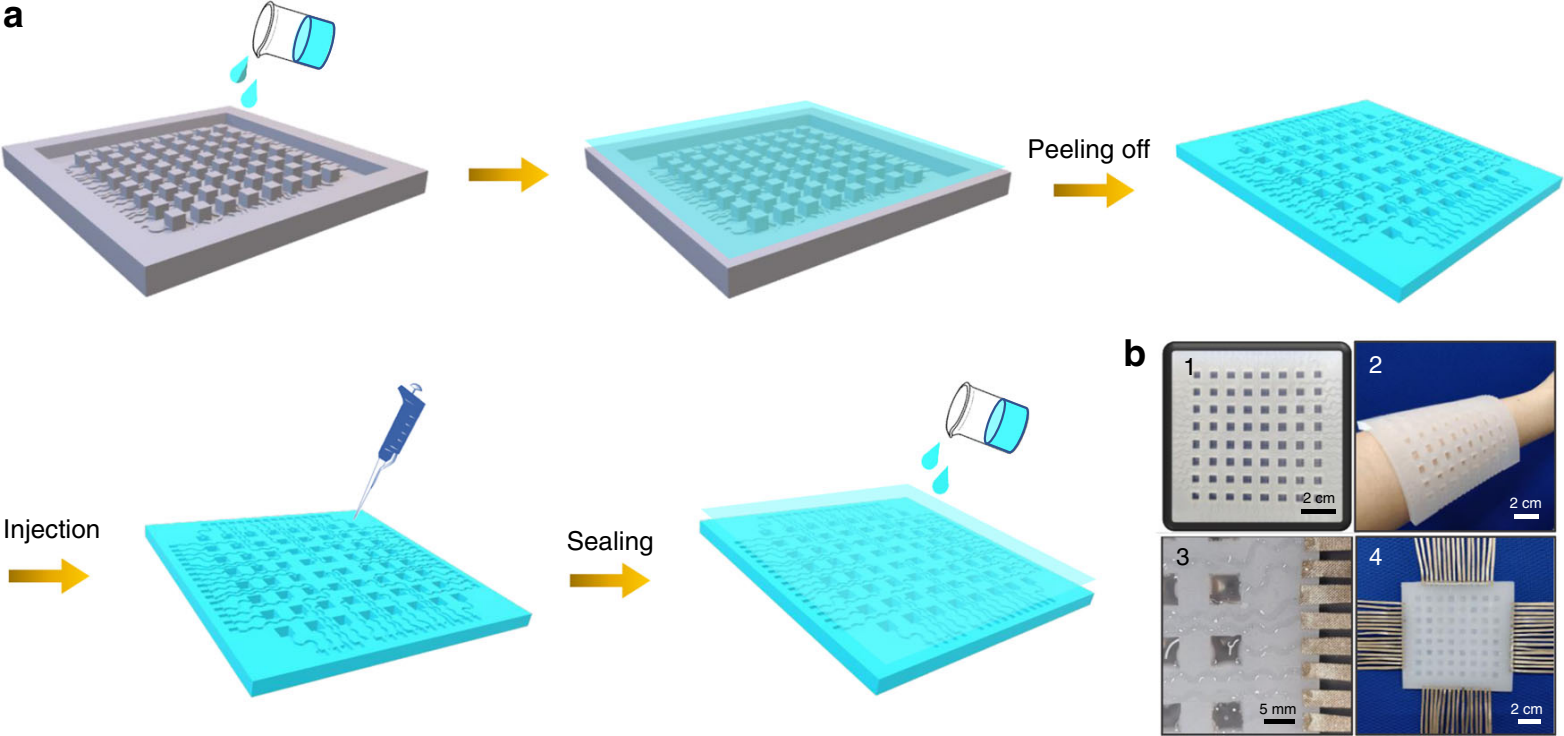
Fabrication flowchart of ISTSA. **a** Schematic illustration of the fabrication process of the ISTSA. **b** Digital photographs of the soft substrate transferred from a 3D template **b**1, **b**2, injection of the conductive gel **b**3, and ISTSA **b**4

The fabrication process of the ISTSA is schematically illustrated in Fig. 1a, and details can be found in the “Materials and methods” section. First, a template with a designed pattern composed of 8-by-8 sensor units and serpentine electrode lines was obtained by three-dimensional (3D) printing (Fig. S1). Each unit has dimensions of 5 mm × 5 mm × 5 mm (length × width × depth). The serpentine structural electrode (width of 1 mm and depth of 0.6 mm) ensures the stretchability of the ISTSA. Then, Ecoflex 00-30 or 00-50 was poured into the template to ensure full coverage. After air-drying at room temperature, a soft and flexible patterned elastomer can be peeled off from the template, leaving cavities in sensor units and serpentine lines (Figs. 1b1 and b2). Accordingly, the mixture of PVA/PEI gel was injected into these cavities, and conductive tape was connected at each end of the serpentine lines (Fig. 1b3). Finally, a thin layer of Ecoflex 00-30 was poured on the plane of the filled elastomer to seal the device. After thoroughly drying in air, the ISTSA (10 cm × 10 cm × 0.6 cm) was obtained (Fig. 1b4) and was ready for the performance test after the device was turned over.

**Fig. 2:**
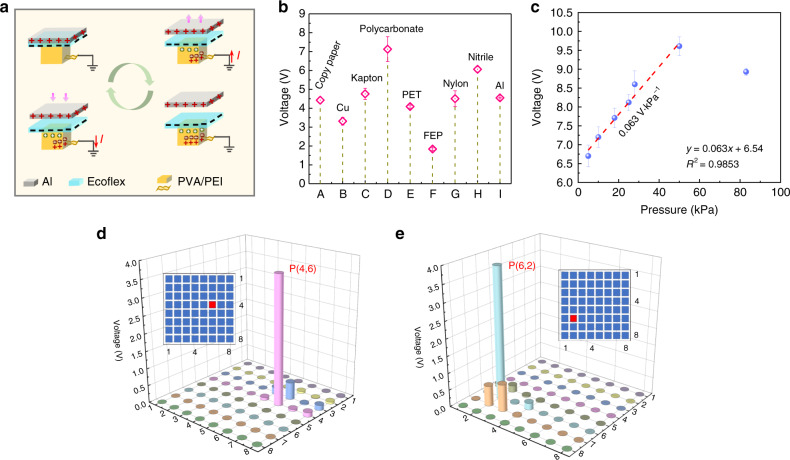
Working mechanism and the output performance of one sensor unit. **a** Working mechanism of the ISTSA based on one sensor unit. **b** The output voltage of one sensor unit in response to various contact materials (contact area of 1 cm^2^) under the same mechanical stimuli. **c** The output voltage of one sensor unit using Al as the contact material (area of 1 cm^2^) as a function of input pressure. **d** and **e** Distribution of output voltage when finger touches one sensor unit as indicated the unit position in red (insets in **d** and **e**). The data acquisition instrument has an internal resistance of 1 MΩ

**Fig. 3:**
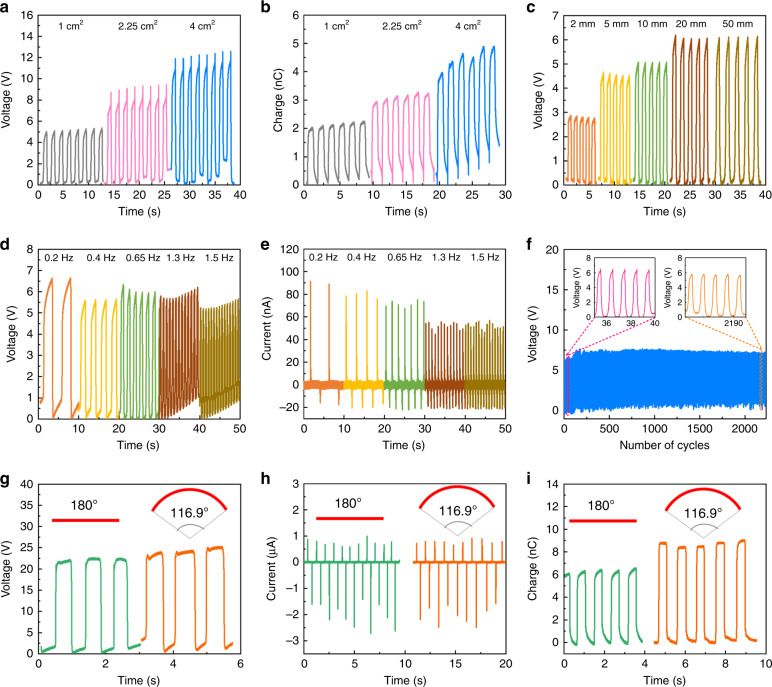
Output characteristics of one sensor unit of the ISTSA in response to various mechanical stimuli. **a** The output voltage and **b** charge when in contact with different contact areas of Al. **c** The output voltage with respect to different displacements of mechanical input (Al as counterpart). **d** The output voltage and **e** current under different frequencies of mechanical input (Al as counterpart). **f** The long-term stability in contact with Al (insets show the output of 35–39 cycles and 2187–2191 cycles). **g** The output voltage, **h** current, and **i** charge of the ISTSA (one unit) when under the flat state (180°) and bending state (116.9°) in response to a finger touch

**Fig. 4:**
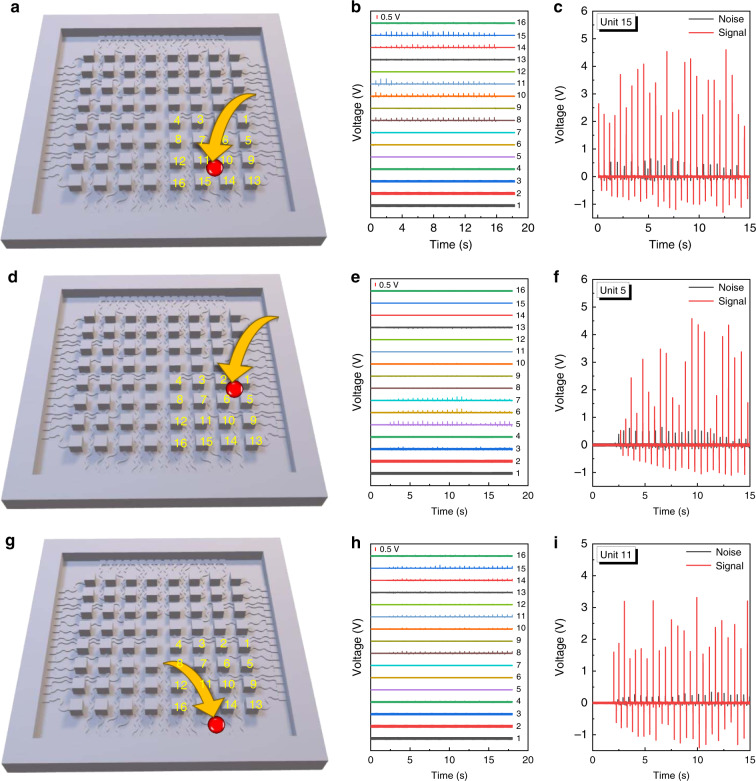
The comparison of the influence of three touch positions on electrode lines to the output characteristics of sensor units. **a** Touch position one (indicated in red circle), **b** real-time output of 1–16 sensor units when a finger touches the red position in **a**, and **c** signal-to-noise comparison of sensor unit 15. Signal: output of unit 15 individually. Noise: output of unit 15 when touching the red position. **d** Touch position two (indicated in red circle), **e** real-time output of 1–16 sensor units when a finger touches the red position in **d**, and **f** signal-to-noise comparison of sensor unit 5. Signal: output of unit 5 individually. Noise: output of unit 5 when touching the red position. **g** Touch position three (indicated in red circle), **h** real-time output of 1–16 sensor units when a finger touches the red position in **g**, and **i** signal-to-noise comparison of sensor unit 11. Signal: output of unit 11 individually. Noise: output of unit 11 when touching the red position

Compared to a previous report with PVA/PEI film as an ionic conductor^38^, in this study, the gel-state PVA/PEI shows a much higher ionic conductivity of 1.53 mS cm^–1^ (Fig. S2), which renders it more efficient as a current collector. The working mechanism of each sensor unit of the ISTSA in the single-electrode mode under a contact-separation cycle is schematically illustrated in Fig. 2a. When an object (taking Al as an example) is fully in contact with one sensor unit, electrification triggers the electrons to be injected from Al to the surface of the unit due to the high electronegativity of silicone rubber. Once Al begins to be withdrawn from the sensor unit, the static negative charge on the silicone rubber will induce the movement of ions in the unit bulk, as well as in the serpentine electrode, leaving positive ions at the interface of the elastomer/electrolyte. Meanwhile, an electrical double layer (EDL) is formed at the conductive tape (metal)/electrolyte interface, with the same number of negative ions. Thus, continuous contact–separation between the sensor unit and contact object will result in a potential difference between the EDL and the ground, and alternating current signals are generated through the external circuit along with the formation of the EDL or neutralization of the positive charges in the EDL.

To demonstrate the output characteristics of the sensor array in terms of various sensing conditions, one individual sensor unit was first taken for investigation. Fig. 2b shows the output voltage of one sensor unit (5 × 5 mm^2^) when in contact with different materials (1 × 1 cm^2^) under the same conditions (velocity of 1 m s^–1^, frequency of 0.65 Hz, displacement of 20 mm), such as copy paper, metal (Cu, Al), Kapton, polycarbonate (PC), polyethylene terephthalate (PET), fluorinated ethylene propylene (FEP), nylon, and nitrile. The output voltage varies greatly in the range of 2–7.1 V, among which the highest output voltage (7.1 V) was reached by contact with PC. The reason is that different materials have distinct electronegativity values due to their intrinsic properties in terms of easy gain or loss of electrons, and according to the triboelectric series, different surface charge densities as well as outputs will be generated upon contact. Thus, when a single sensor unit is in contact with various materials, different magnitudes of electrical responses are yielded. The results demonstrate the potential application of the sensor array in response to various sensing environments. To investigate the pressure sensitivity of the ISTSA, the voltage response while gradually increasing the applied pressure on one sensor unit was recorded. It is notable that the output voltage showed a positive linear correlation with increasing contact pressure in the range of 5–50 kPa (Fig. 2c), with a pressure sensitivity of 0.063 V kPa^–1^, owing to the increased surface contact area of the unit. However, further increasing the pressure to 80 kPa caused severe compression of the sensor unit; thus, the regain of the sensor unit together with the retraction of the contacting object may disturb the surface charge density, leading to a decrease in output. Compared to a previously reported multilayer sensor matrix^31^, the ISTSA exhibits a comparable pressure sensitivity and sensing range. Additionally, the voltage responses of one sensor unit of ISTSA under different input pressures with respect to those of other contacting materials, such as PC and FEP, were investigated, as shown in Fig. S3, where the pressure sensitivity is 0.0294 V kPa^–1^ for PC in a sensing range of 5–55 kPa and 0.0318 V kPa^–1^ for FEP in the range of 5–75 kPa.

As is known, the electrostatic charge induced on the elastomer surface inevitably interferes with the sensing behavior of sensor units^45^. To investigate how large the influence of each sensor unit is on the adjacent units, the real-time output voltage of other sensor units was recorded at the same time when one unit was touched. Fig. 2d, e indicates the output distributions in response to touching positions of P (4,6) (red square in inset of Fig. 2d) and P (6,2) (red square in inset of Fig. 2e). It is evident that the adjacent units also had electrical responses when P (4,6) and P (6,2) were touched by a finger. Nevertheless, the crosstalk (0.01–0.55 V) was relatively small in comparison with the output of the triggered sensor unit (>3.7 V) (Supporting Information, Video 1), demonstrating the reliability of the ISTSA to precisely identify the touching position of a contact object. For another type of crosstalk arising from the electrodes, a detailed discussion will be conducted later.

To further investigate the electrical output characteristics of one sensor unit of the ISTSA in response to various mechanical stimuli, different contact areas, displacements, and frequencies of mechanical input were evaluated when in contact with Al, as shown in Fig. 3a–e (setup shown in Fig. S4). The output voltage increases from 5 to 12 V when the contact area of Al increases from 1 to 4 cm^2^ under a pressure of ~50 kPa (Fig. 3a), and the output charge follows the same trend, rising from 2 to 4.5 nC (Fig. 3b). The results agreed well with previous studies showing that an increase in the surface area results in an enhanced surface charge and output voltage^46^. Additionally, the output of the triboelectric ISTSA has a relationship with the displacement between the contact object and the sensor unit. Figure 3c presents the output voltage, which gradually increases from ~3 to 6 V as the displacement increases from 2 to 20 mm. A further increase to 50 mm brings no enhancement of the output voltage, which means that electrons/ions in the sensor unit have reached an equilibrium state at the displacement of 20 mm. Moreover, it is found that the mechanical input frequency also influences the output voltage and current signals at a fixed 20-mm displacement (Fig. 3d, e). A lower frequency (0.2 Hz) tends to exhibit full contact between Al and the sensor unit, yielding a higher output voltage of 6.6 V and current of 91 nA, while a higher frequency (1.3–1.5 Hz) leads to a certain decrease in the contact area, generating a relatively lower output voltage of 5.2–5.7 V and current of 55 nA. Thus, a low-frequency mechanical stimulus is suggested for better sensing output of ISTSA. In addition, the long-term stability of the sensor unit was tested, as shown in Fig. 3f. It is noted that the output voltage of one sensor unit in contact with Al has no obvious drop even after 2250 contact cycles under a frequency of 1 Hz, demonstrating the potential for long-term-sensing applications.

Due to the unique design of the serpentine electrodes as well as the soft-integrated elastomer profile, the ISTSA can feasibly perform sensing under the bending state. Fig. 3g–i presents a comparison of the output voltage, current, and charge of one sensor unit in a flat state (180°) and a bending state (116.9°) (Fig. S5) upon finger touch. The overall output is much higher than that in Fig. 3a–c because human skin (finger) is more positive than Al according to the triboelectric series^47^. As seen, the ISTSA generates output voltage, current, and charge values of 24 V, 2.5 μA, and 9 nC, respectively, when bent at 116.9°, while in the flat state, the output is 22.5 V, 2.6 μA, and 6.5 nC. The comparable or even higher output under the bending state is mainly due to the enlarged surface area of the sensor unit during bending; thus, more surface charges can be generated. This result demonstrates the promising application of the ISTSA when the sensing circumstance involves a certain curvature, which indicates an advantage over other touch sensor arrays with a hard substrate.

As discussed above, crosstalk between the sensor units and the electrode lines is another key issue that needs to be circumvented in order to have a sensor array capable of precise sensing. Commonly, researchers have adopted shielding layers to cover the electrode part to reduce or minimize the crosstalk. For example, a conductive nickel-deposited fabric layer was employed with a screening effect to reduce the crosstalk^45^. However, given that adopting more layers complicates the fabrication process, the mismatch of the elastic modulus between different layers may cause a relative shift when the device is in a bent state, which will affect the sensitivity of the sensor array. Thus, in our design, without the addition of other layers, we present a facile fabrication approach that can adequately suppress the crosstalk simply by introducing a height difference between the sensor unit and electrode, with 5 mm-height sensor units and 0.6-mm height electrode lines. The height difference, consequently, is larger than eight-fold. For a clear demonstration, three different positions (Fig. 4a, d, g) were checked, and the real-time output signals of 16 adjacent sensor units were recorded at the same time when one position was touched by a finger with a touching force of 1.2–1.5 N and frequency of ~1.7 Hz. (Fig. 4b, e, h). Notably, among the three positions, the first touching point (Fig. 4a) showed a relatively higher impact on the adjacent 16 sensor units, leading to five sensor units with noise signal generation (Fig. 4b), and the second touching point (Fig. 4d) affected three sensor units. Additionally, at the edge of the sensor array (the third touching point, Fig. 4g), there was a certain impact on the sensor units but with negligible noise (Fig. 4h). Furthermore, the sensor unit with the highest magnitude of signal/noise output during triggering by touching positions was selected from Fig. 4b, e, and h and compared with its own signal output, as plotted in Fig. 4c, f, and i, respectively. In this case, we can make a thorough comparison of the signal-to-noise ratio of one individual sensor unit. As calculated, the signal-to-noise ratios of sensor unit 15, unit 5, and unit 11 are 8.1, 8.2, and 8.5, respectively. These values are comparable to the height difference mentioned earlier. The results indicate a good differentiation of the signal to the noise and the capability of the ISTSA for valid sensing, demonstrating an effective way to reduce the crosstalk of the electrode lines.

**Fig. 5:**
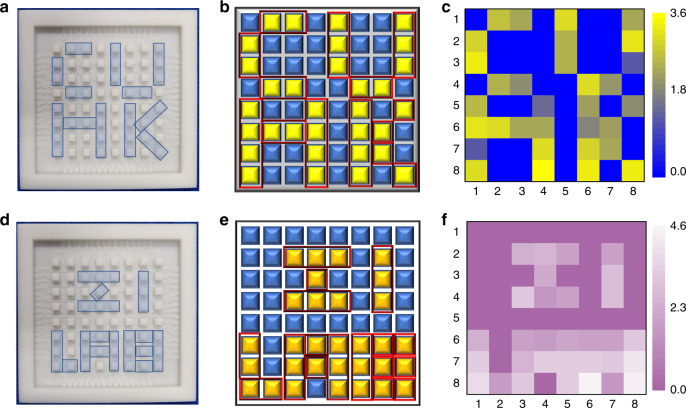
Electrical responses of the ISTSA when external forces are applied in designed patterns. **a** Illustration of the touch pattern “CUHK”. **b** Touching points of the pattern “CUHK” on the ISTSA. **c** Demonstration of the corresponding electrical responses of the touched pattern “CUHK”. **d** Illustration of the touch pattern “ZI LAB”. **e** Touching points of the pattern “ZI LAB” on the ISTSA. **f** Demonstration of the corresponding electrical responses of the touched pattern “ZI LAB”

Further, to demonstrate the uniformity and sensitivity of the sensor array in response to a finger touch, two different patterns depicted as “CUHK” and “ZI LAB”, as shown in Fig. 5a and d, respectively, were touched in sequence by fingers on two 8 × 8 sensor arrays. Accordingly, Fig. 5b and e illustrate the associated patterns expected on the two ISTSAs, and the actual electrical responses generated by finger touching are ~1.2–3.6 V (Fig. 5c) for the ISTSA with a “CUHK” pattern and 1.6–4.6 V (Fig. 5f) for the other ISTSA with a “ZI LAB” pattern. The small fluctuations in the voltage responses indicate not only the uniformity of the sensor units within one ISTSA but also the reproducibility between the ISTSAs. In addition, the output voltage signals of eight sensor units of one ISTSA in a diagonal position are shown in Fig. S6. The difference in the responses is mainly due to the different lengths (resistances) of the serpentine lines.

Finally, we demonstrate a practical application of the ISTSA by lighting up an LED array through gentle touching of the corresponding sensor unit by a finger. Three sensor units, as examples, with their associated LED lighting at the positions of P (6,1), P (2,3), and P (2,6), are shown in Fig. S7 (Supporting Information, Video 2). The real-time LED lighting of the ISTSA by finger touching shows its promising application in position tracking.

## Conclusion

In summary, we present a fully polymer-integrated triboelectric tactile sensor array (8 × 8) without a metal electrode through a facile and low-cost fabrication process. The sensor unit shows a pressure sensitivity of 0.063 V kPa^–1^ in the range of 5–50 kPa and is sensitive to various contact objects as well as capable of sensing under a bending state. The crosstalk arising from the electrodes is successfully suppressed by introducing an eight-fold height difference between the sensor units and electrodes, resulting in a signal-to-noise ratio larger than eight. In addition, small fluctuations in the voltage responses in pattern mapping by finger touching indicate the uniformity of the sensor array. Finally, the self-powered ISTSA is demonstrated for real-time LED lighting by finger touching, which shows its promising application in position tracking, human–machine interfaces, and wearable electronics.

## Materials and methods

### **F**abrication of the soft integrated tactile sensor array (ISTSA)

The ISTSA was fabricated by a template-assisted method (Fig. 1). In detail, a template with patterns was 3D printed with detailed dimensions, and the digital photograph is shown in Fig. S1. Then, Ecoflex 00-30/00-50 (Smooth-on, USA) rubbers were mixed thoroughly with A:B by the same weight and poured into the template to ensure full coverage, followed by degassing under vacuum, and were dried at room temperature. Then, a flexible Ecoflex rubber mold can be peeled off from the template. The second step was to assemble the electrode, where instead of a metal electrode, an ionic conductor composed of a mixture of PVA (Mowiol^®^ 10-98, *M*_w_: ~61,000, Sigma-Aldrich) and PEI (*M*_w_: ~25,000, Sigma-Aldrich) was employed using a modified recipe as reported previously^38^. The volume ratio of PVA to PEI was 10 to 1 to enhance the ionic conductivity. Accordingly, the mixture was injected into the cavities of the mold (both sensor units and serpentine lines). Conductive tape was connected at each end of the serpentine lines for electrical measurement. The last step was sealing the ISTSA. The well-mixed Ecoflex 00-30 (parts A and B with the same weight ratio) was degassed under vacuum in advance and poured on the plane of the mold to fully cover the sensor units and serpentine lines and left to dry in air at room temperature. Consequently, an ISTSA was finally obtained.

### Characterization and measurement

The ionic conductivity of the PVA/PEI was measured by a Eutech PC 700 Meter (Thermo Scientific). The electrical output (voltage, current, and charge) of a single sensor unit was measured by a Keithley 6514 programmable electrometer. The real-time signal sensing was recorded by a DAQ system (PowerLab 16/35, ADInstruments) equipped with 16 input channels with an internal resistance of 1 MΩ.

## Supplementary information

Supplemental material

Supporting Video 1

Supporting Video 2

## References

[1] Yu XG (2019). Skin-integrated wireless haptic interfaces for virtual and augmented reality. Nature.

[2] Wang SH (2018). Skin electronics from scalable fabrication of an intrinsically stretchable transistor array. Nature.

[3] Han M (2019). Three-dimensional piezoelectric polymer microsystems for vibrational energy harvesting, robotic interfaces and biomedical implants. Nat. Electron..

[4] Lee, Y. et al. Mimicking human and biological skins for multifunctional skin electronics. *Adv*. *Funct*. *Mater*. 1904523 (2019).

[5] Miyamoto A (2017). Inflammation-free, gas-permeable, lightweight, stretchable on-skin electronics with nanomeshes. Nat. Nanotechnol..

[6] Shi M (2016). Self-powered analogue smart skin. ACS Nano.

[7] Ankanahalli Shankaregowda S (2019). Single-electrode triboelectric nanogenerator based on economical graphite coated paper for harvesting waste environmental energy. Nano Energy.

[8] Ankanahalli Shankaregowda S (2019). Dry-coated graphite onto sandpaper for triboelectric nanogenerator as an active power source for portable electronics. Nanomaterials.

[9] Shi M (2017). Self-powered wireless smart patch for healthcare monitoring. Nano Energy.

[10] Wu C (2020). Self-powered tactile sensor with learning and memory. ACS Nano.

[11] Zang YP, Zhang FJ, Di CA, Zhu DB (2015). Advances of flexible pressure sensors toward artificial intelligence and health care applications. Mater. Horiz..

[12] Song Y (2017). Highly compressible integrated supercapacitor-piezoresistance-sensor system with CNT-PDMS sponge for health monitoring. Small.

[13] Chen HT (2018). Hybrid porous micro structured finger skin inspired self-powered electronic skin system for pressure sensing and sliding detection. Nano Energy.

[14] Choi W (2019). Stretchable triboelectric multimodal tactile interface simultaneously recognizing various dynamic body motions. Nano Energy.

[15] Song Y (2019). High-efficiency self-charging smart bracelet for portable electronics. Nano Energy.

[16] Li JP (2014). Healable capacitive touch screen sensors based on transparent composite electrodes comprising silver nanowires and a furan/maleimide Diels–Alder cycloaddition polymer. ACS Nano.

[17] Nie BQ, Li RY, Cao J, Brandt JD, Pan TR (2015). Flexible transparent iontronic film for interfacial capacitive pressure sensing. Adv. Mater..

[18] Lin MF, Xiong JQ, Wang JX, Parida K, Lee PS (2018). Core–shell nanofiber mats for tactile pressure sensor and nanogenerator applications. Nano Energy.

[19] Shi JD (2018). Multiscale hierarchical design of a flexible piezoresistive pressure sensor with high sensitivity and wide linearity range. Small.

[20] Pan LJ (2014). An ultra-sensitive resistive pressure sensor based on hollow-sphere microstructure induced elasticity in conducting polymer film. Nat. Commun..

[21] Wang T (2018). A Self-healable, highly stretchable, and solution processable conductive polymer composite for ultrasensitive strain and pressure sensing. Adv. Funct. Mater.

[22] Liu YM (2019). Skin-integrated graphene-embedded lead zirconate titanate rubber for energy harvesting and mechanical sensing. Adv. Mater. Technol..

[23] Yu XE (2018). Needle-shaped ultrathin piezoelectric microsystem for guided tissue targeting via mechanical sensing. Nat. Biomed. Eng..

[24] Zi YL (2015). Triboelectric-pyroelectric-piezoelectric hybrid cell for high-efficiency energy-harvesting and self-powered sensing. Adv. Mater..

[25] Yao HB (2013). A flexible and highly pressure-sensitive graphene-polyurethane sponge based on fractured microstructure design. Adv. Mater..

[26] Wang H (2017). Self-powered dual-mode amenity sensor based on the water-air triboelectric nanogenerator. ACS Nano.

[27] Liu YM (2020). Thin, skin-integrated, stretchable triboelectric nanogenerators for tactile sensing. Adv. Electron. Mater.

[28] Xi Y (2017). Multifunctional TENG for blue energy scavenging and self-powered wind-speed sensor. Adv. Energy Mater..

[29] Tao J (2019). Self-powered tactile sensor array systems based on the triboelectric effect. Adv. Funct. Mater..

[30] Lee Y (2019). Graphene-based stretchable/wearable self-powered touch sensor. Nano Energy.

[31] Wang XD (2016). Self-powered high-resolution and pressure-sensitive triboelectric sensor matrix for real-time tactile mapping. Adv. Mater..

[32] Wang XD (2018). A highly stretchable transparent self-powered triboelectric tactile sensor with metallized nanofibers for wearable electronics. Adv. Mater..

[33] Kim CC, Lee HH, Oh KH, Sun JY (2016). Highly stretchable, transparent ionic touch panel. Science.

[34] Zhao GR (2019). Transparent and stretchable triboelectric nanogenerator for self-powered tactile sensing. Nano Energy.

[35] Lei ZY, Wang QK, Sun ST, Zhu WC, Wu PY (2017). A Bioinspired mineral hydrogel as a self-healable, mechanically adaptable ionic skin for highly sensitive pressure sensing. Adv. Mater.

[36] Liu SJ, Li L (2017). Ultrastretchable and self-healing double-network hydrogel for 3D printing and strain sensor. ACS Appl. Mater. Interfaces.

[37] Wang LY, Daoud WA (2019). Hybrid conductive hydrogels for washable human motion energy harvester and self-powered temperature-stress dual sensor. Nano Energy.

[38] Wang LY, Daoud WA (2019). Highly flexible and transparent polyionic-skin triboelectric nanogenerator for biomechanical motion harvesting. Adv. Energy Mater..

[39] Pu X (2017). Ultrastretchable, transparent triboelectric nanogenerator as electronic skin for biomechanical energy harvesting and tactile sensing. Sci. Adv.

[40] Hines L, Petersen K, Lum GZ, Sitti M (2017). Soft actuators for small-scale robotics. Adv. Mater..

[41] Yang Y (2013). Human skin based triboelectric nanogenerators for harvesting biomechanical energy and as self-powered active tactile sensor system. ACS Nano.

[42] Yi F (2014). Self-powered trajectory, velocity, and acceleration tracking of a moving object/body using a triboelectric sensor. Adv. Funct. Mater..

[43] Yao G (2020). Bioinspired triboelectric nanogenerators as self-powered electronic skin for robotic tactile sensing. Adv. Funct. Mater.

[44] Ren ZW (2018). Fully elastic and metal-free tactile sensors for detecting both normal and tangential forces based on triboelectric nanogenerators. Adv. Funct. Mater.

[45] Zhu XX (2017). Triboelectrification-enabled touch sensing for self-powered position mapping and dynamic tracking by a flexible and area-scalable sensor array. Nano Energy.

[46] Chun J (2015). Mesoporous pores impregnated with Au nanoparticles as effective dielectrics for enhancing triboelectric nanogenerator performance in harsh environments. Energy Environ. Sci..

[47] Wang ZL (2013). Triboelectric nanogenerators as new energy technology for self-powered systems and as active mechanical and chemical sensors. ACS Nano.

