# Supplementary material for: A metal-electrode-free, fully integrated, soft triboelectric sensor array for self-powered tactile sensing
Source: Microsyst Nanoeng. 2020 Aug 10;6:59. doi: 10.1038/s41378-020-0154-2 (PMC8433331; doi:10.1038/s41378-020-0154-2)
Supplement: Supplementary file 1 — Supplemental material [file 41378_2020_154_MOESM1_ESM.docx]

**Supporting Information**

**A Metal-Electrode-Free, Fully-Integrated, Soft Triboelectric Sensor Array for Self-Powered Tactile Sensing**

Lingyun Wang^1⊥^, Yiming Liu^2⊥^, Qing Liu^3^, Yuyan Zhu^3^, Haoyu Wang^1^, Zhaoqian Xie^4^, Xinge Yu^2^*, Yunlong Zi^1^*

*1 Department of Mechanical and Automation Engineering, The Chinese University of Hong Kong, Shatin, N.T., Hong Kong SAR, China.*

2 Department of Biomedical Engineering, City University of Hong Kong, Kowloon, Hong Kong SAR, China.

3 Department of Applied Biology and Chemical Technology, The Hong Kong Polytechnic University, Hung Hom, Hong Kong SAR, China.

4 State Key Laboratory of Structural Analysis for Industrial Equipment, International Research Center for Computational Mechanics, Department of Engineering Mechanics, Dalian University of Technology, Dalian, 116024, China

E-mail: [xingeyu@cityu.edu.hk](mailto:xingeyu@cityu.edu.hk) (XY) and [ylzi@cuhk.edu.hk](mailto:ylzi@cuhk.edu.hk) (YZ)


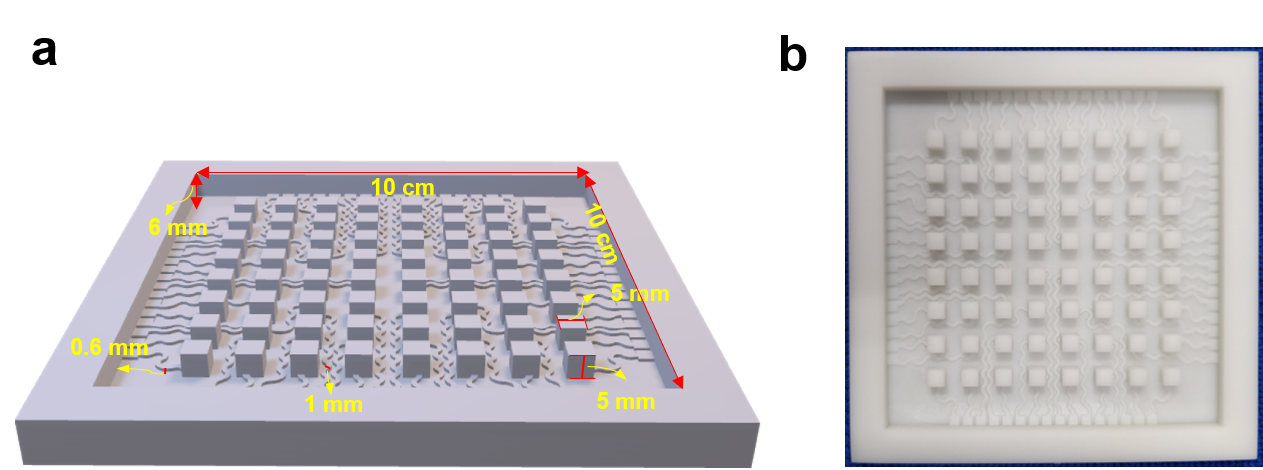


**Figure S1**. (a) Detail dimensions of three-dimensional (3D) printed template. (b) Digital photograph of the template.


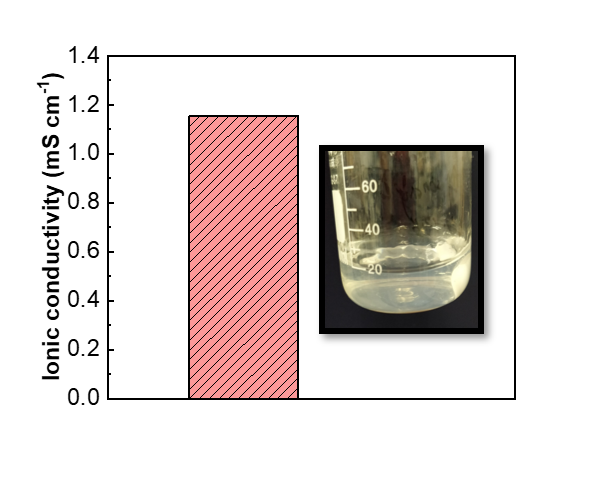


**Figure S2**. Ionic conductivity of PVA/PEI gel. The inset shows its digital photograph.


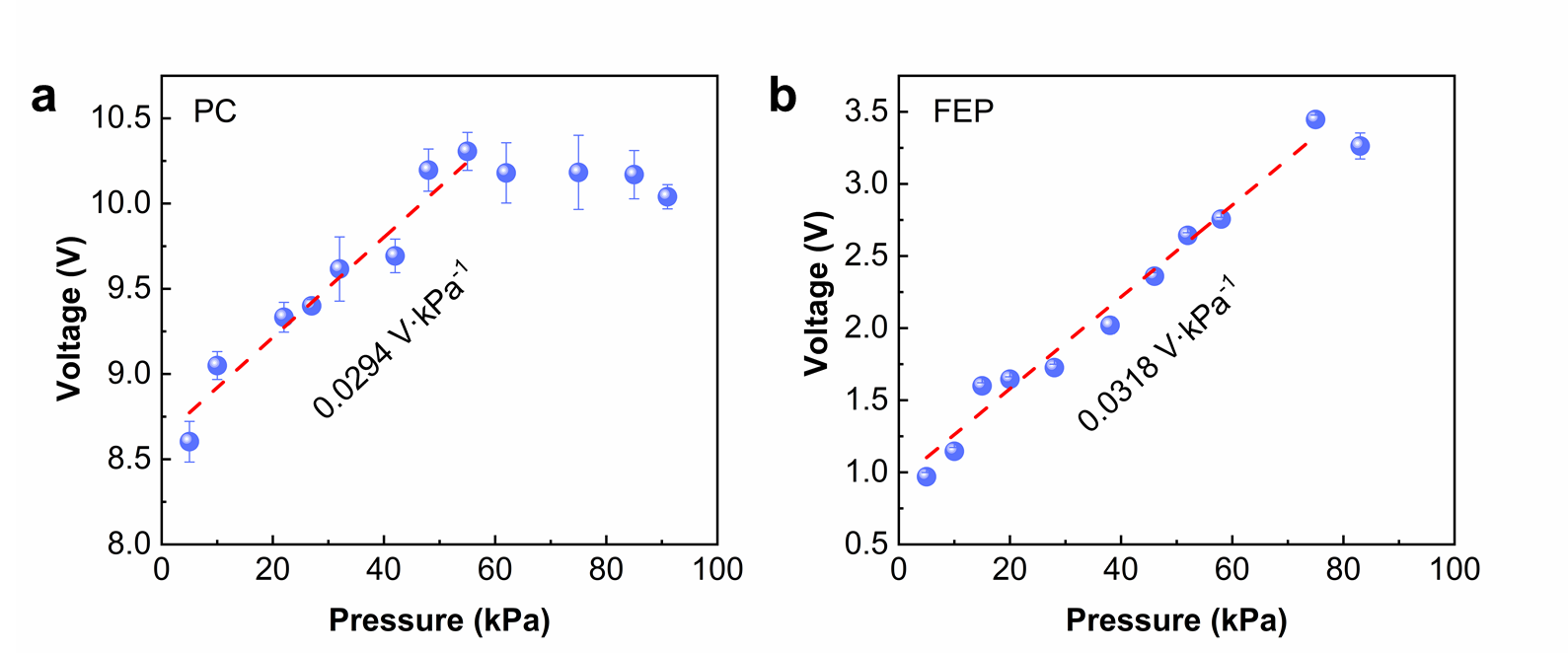


**Figure S3**. The output voltage of (a) PC and (b) FEP as a function of pressure.


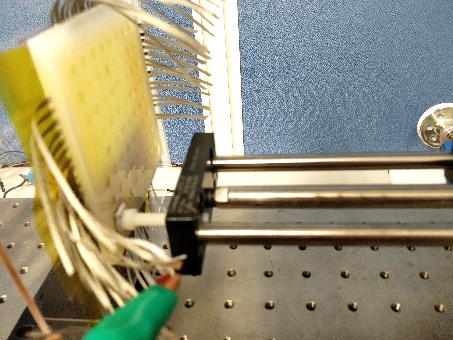


**Figure S4.** Demonstration of one sensor unit in contact with Al or other materials.


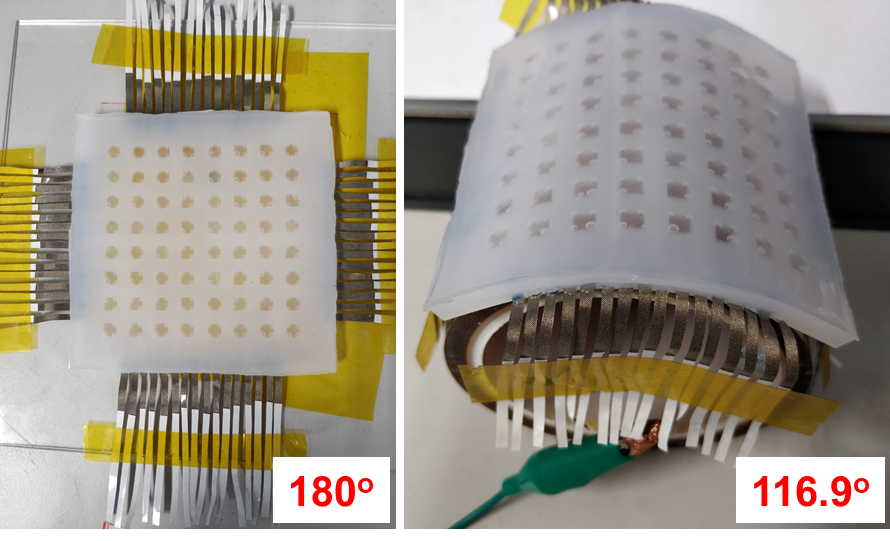


**Figure S5.** Demonstration of ISTSA sensing at a flat state (180^o^) and a bending state (116.9^o^).


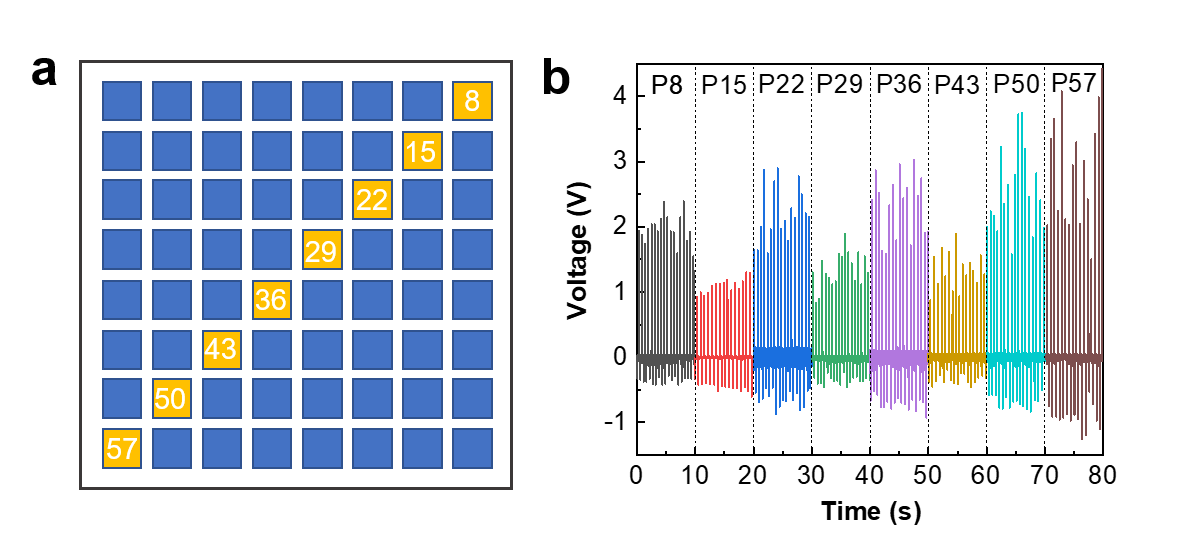


**Figure S6**. (a) Touching positions on ISTSA in a diagonal direction. (b) Voltage responses of the corresponding sensor units in (a).


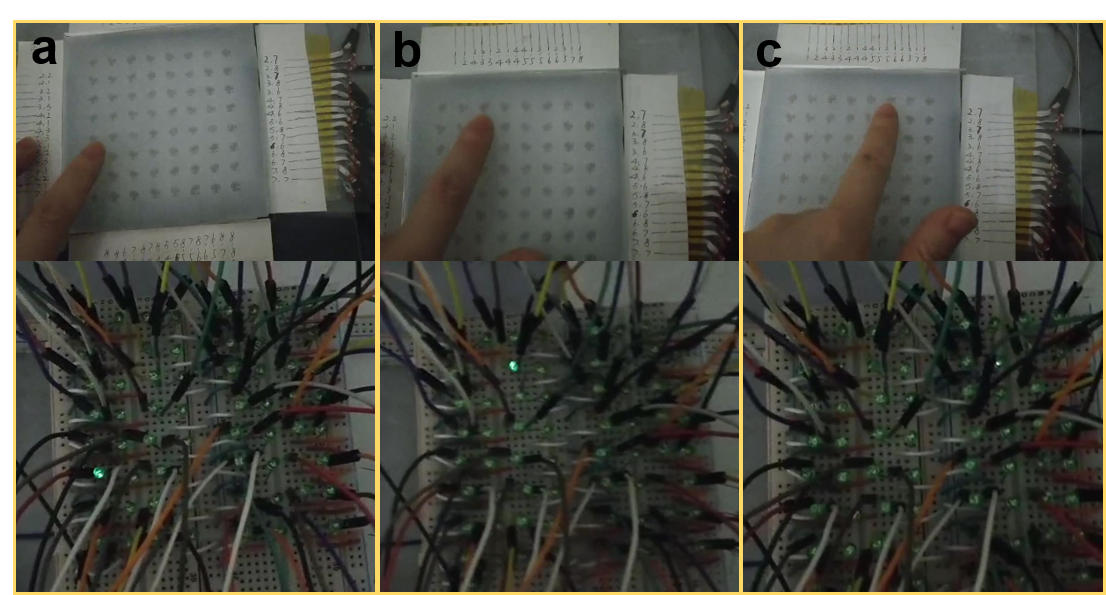


**Figure S7.** Demonstration of gentle touching the sensor unit of ISTSA by finger to trigger corresponding LED lighting. (a) P (6,1). (b) P (2, 3). (c) P (2, 6).
